# Chronic simian immunodeficiency virus infection is associated with contrasting phenotypes of dysfunctional Bcl6^+^ germinal center B cells or Bcl6^−^Bcl2^+^ non‐germinal center B cells

**DOI:** 10.1111/jcmm.13844

**Published:** 2018-09-06

**Authors:** Olusegun O. Onabajo, Mark G. Lewis, Joseph J. Mattapallil

**Affiliations:** ^1^ F. Edward Hébert School of Medicine Uniformed Services University Bethesda Maryland; ^2^ Bioqual Inc. Kensington Maryland; ^3^ Present address: Laboratory of Translational Genomics Division of Cancer Epidemiology and Genetics National Cancer Institute National Institutes of Health Bethesda Maryland

**Keywords:** B cells, Bcl2, Bcl6, germinal center, human immunodeficiency virus, IL‐21, IL‐21R, simian immunodeficiency virus, T follicular helper cells

## Abstract

Human immunodeficiency virus (HIV) infection is characterized by dysfunctional B cell responses. Here we show that chronic simian immunodeficiency virus (SIV) infection is characterized by an expansion of either lymph node germinal center (GC) B cells that co‐express Bcl6, Ki‐67 and IL‐21R and correlate with expanded T follicular helper (Tfh) cells or B cells that lack Bcl6, Ki‐67 and IL‐21R but express high levels of anti‐apoptotic Bcl2 that negatively correlate with Tfh cells. The lack of Tfh cells likely contributes to persistence of dysfunctional non‐proliferating B cells during chronic infection. These findings have implications for protective immunity in HIV‐infected individuals who harbour low frequencies of Tfh cells.

## 1 BACKGROUND

B cell dysfunction has been well documented during human immunodeficiency virus (HIV) and simian immunodeficiency virus (SIV) infections and is characterized by hypergammaglobulinemia or B cell exhaustion.^1^ Numerous studies have implicated a role for expanded T follicular helper (Tfh) cells in driving the expansion of B cells in the germinal centers.^2^, ^3^, ^4^ On the other hand, lack of help from Tfh cells has been reported to impair B cell immune responses during HIV infection.^5^

Studies have shown that Bcl6 expression in T cells drives the differentiation of Tfh cells, whereas its expression is necessary for the maintenance of germinal center (GC) B cells.^6^, ^7^ In contrast, Bcl6 expression suppresses the expression of Bcl2^8^, an anti‐apoptotic protein that has been implicated in the development of follicular lymphomas. Bcl2 is generally not expressed in GC B cells but inducing their expression can prevent apoptosis in GC B cells.^9^ The contrasting roles of these two transcription factors during HIV infection are not well defined and may be important in understanding the mechanisms associated with B cell dysfunction during HIV infection. Using the rhesus macaque model for chronic SIV infection, we examined the expression of Bcl6 and Bcl2 in lymph node B cells and correlated changes in these subsets with Tfh cells. We observed two distinct groups of SIV infected animals with lymph node (LN) B cells that expressed either high levels of Bcl6 (SIV^+^Bcl6^hi^) or lacked Bcl6 expression (SIV^+^Bcl6^lo^). The lack of Bcl6 in B cells was accompanied by significantly lower frequencies of Tfh cells and a higher expression of Bcl2. Given the anti‐apoptotic nature of Bcl2, it likely contributes to the expansion and persistence of dysfunctional B cells that display a hypo‐proliferative and IL‐21R^−^ phenotype. These finding provide additional insights into B cell dysfunction observed during chronic HIV infection.

## 2 MATERIALS AND METHODS

### 2.1 Animals and samples

Archival mesenteric lymph nodes (LN) and plasma samples that were collected at necropsy from uninfected (n = 6) and SIVmac251 infected (n = 10) rhesus macaques (*Macaca mulatta*) were used in this study. All animals were chronically infected for over 6 months at the time of sample collection and housed at Bioqual, Inc. in accordance with the recommendations of the Association for Assessment and Accreditation of Laboratory Animal Care International Standards. The animals were sero‐negative for SIV, simian retrovirus (SRV) and simian T cell leukaemia virus (STLV) type‐1.

### 2.2 Antibodies and flow cytometry

Isolated cells were labelled with combinations of anti‐CD3, CD4, CD28, CD95, PD‐1, Bcl6, ICOS, Bcl2, IL‐21R, CD20, IgG, IgM and Ki‐67 and analysed by flow cytometry. The expression of Ki‐67, Bcl6 and Bcl2 expression was determined by Intracellular staining using the eBiosciences Fix/Perm kit. Labelled cells were fixed with 0.5% Paraformaldehyde and analysed using a Becton Dickinson LSR II flow cytometer.

### 2.3 Data analysis

Flow cytometric data was analysed using FlowJo version 9.8 (Tree Star, Inc., Ashland, OR). Statistical differences between groups were determined using one‐way ANOVA and differences within each group were determined by post hoc analysis using Fisher's LSD multiple comparisons test. Linear regression analysis was performed to determine line of fit, and correlations were derived using Spearman's correlation. A *P* < 0.05 was considered significant. Error bars represent standard error.

## 3 RESULTS

### 3.1 Differential expression of Bcl6 and Bcl2 on lymph node B cells during chronic SIV infection

**Figure 1:**
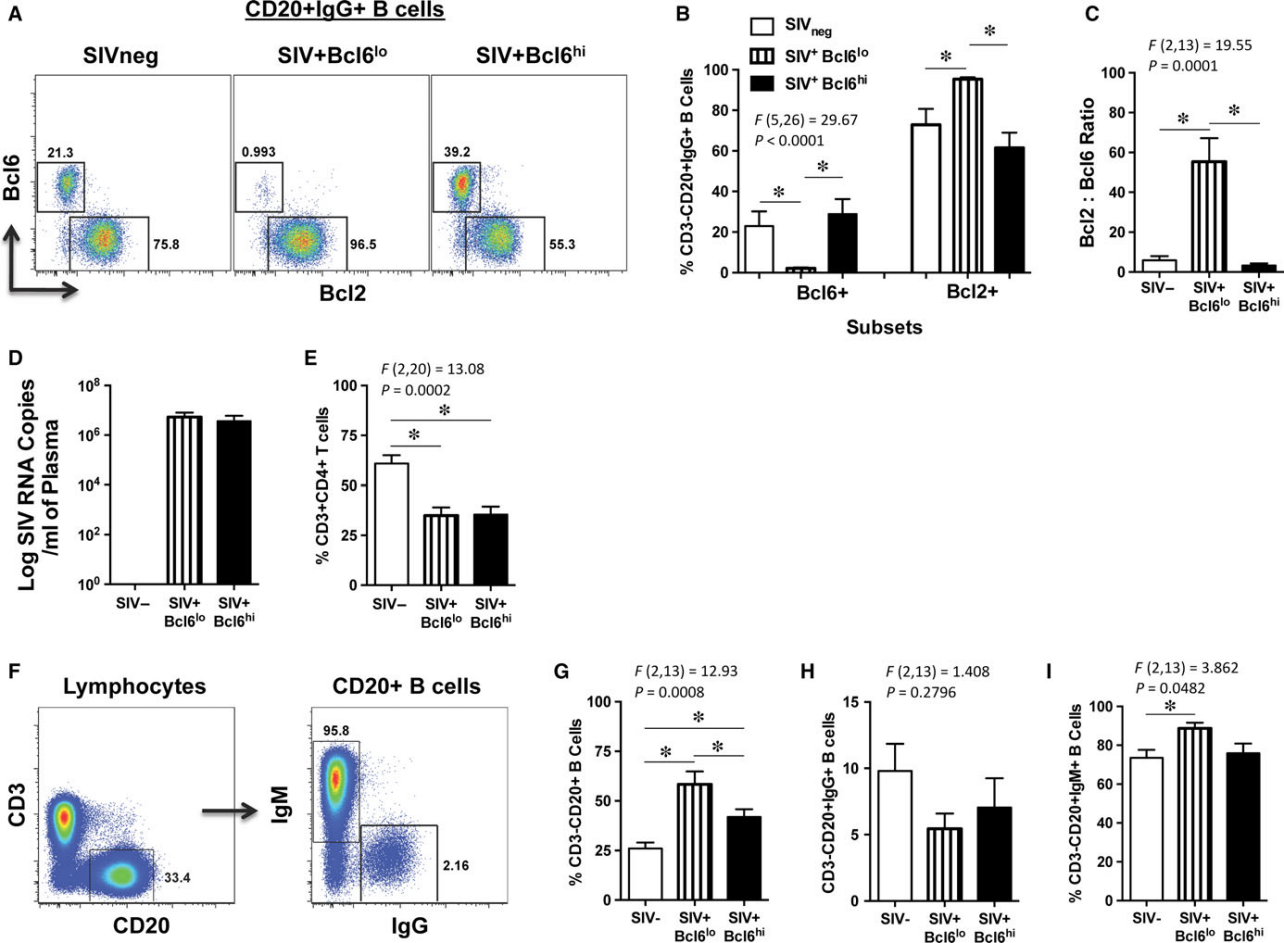
Differential expression of Bcl6 and Bcl2 on Lymph node IgG^+^ B cells during chronic SIV infection. (A) Representative dot plot showing the expression of Bcl2 and Bcl6 on CD20^+^IgG^+^ B cells. (B) Frequency of Bcl6 and Bcl2 expressing CD3‐CD20 + IgG+ B cells in lymph node, (C) ratio of Bcl2: Bcl6, (D) plasma viral loads, (E) % of CD3^+^CD4^+^ T cells from SIV_neg_ (n = 6), SIV^+^Bcl6^lo^ (n = 5) and SIV^+^Bcl6^hi^ (n = 5) groups of animals. (F) Representative dot plot showing the gating strategy used to identify CD3^‐^CD20^+^IgG and IgM^+^ B cells. Frequency of (G) total CD3^‐^CD20^+^, (H) CD3^‐^CD20^+^IgG^+^ and (I) CD3^‐^CD20^+^IgM^+^ B cells in lymph nodes from SIV_neg_ (n = 6), SIV^+^Bcl6^lo^ (n = 5) and SIV^+^Bcl6^hi^ (n = 5) groups of animals

Studies have shown that B cells that participate in GC reaction co‐express high levels of Bcl6,^6^, ^10^ whereas others have reported increased expression of anti‐apoptotic Bcl2 on developmentally blocked GC B cells.^11^ To assess if expression of Bcl6 and Bcl2 was altered during SIV infection, we examined their expression on B cells in the LN from chronically infected animals and compared them to uninfected animals. Our results showed that Bcl6 was differentially expressed in chronically infected animals (Figure 1A) with one group of animals expressing high levels of Bcl6 (SIV^+^Bcl6^hi^) on CD20^+^IgG^+^ B cells that did not significantly differ from uninfected animals, whereas the other group of animals lacked Bcl6 expression (SIV^+^Bcl6^lo^). Interestingly, SIV^+^Bcl6^lo^ group of animals expressed significantly higher levels of Bcl2 (Figure 1B) as compared to the other groups; the Bcl2: Bcl6 ratio was significantly higher in SIV^+^Bcl6^lo^ group of SIV‐infected animals (Figure 1C).

The plasma viral loads (Figure 1D) and the frequency of CD4 T cells (Figure 1E) did not differ significantly between SIV^+^Bcl6^hi^ and SIV^+^Bcl6^lo^ groups of animals. There was, however, a significant difference in the frequency of total B cells between the three groups of animals. Total B cells were discriminated based on the expression of CD20 on CD3^‐^ lymphocytes (Figure 1F). SIV^+^Bcl6^lo^ group of animals were found to have a significantly higher prevalence of CD20^+^ B cells as compared to both uninfected and SIV^+^Bcl6^hi^ group of animals (Figure 1G). There was, however, no significant difference in frequency of IgG^+^ B cells between the groups (Figure 1H) likely because of variation between animals though the SIV^+^Bcl6^lo^ group of animals had a higher prevalence of IgM^+^ B cells as compared to uninfected animals (Figure 1I).

### 3.2 High levels of Bcl2 expression negatively correlate with IL‐21R and Ki‐67 expression on LN B cells

**Figure 2:**
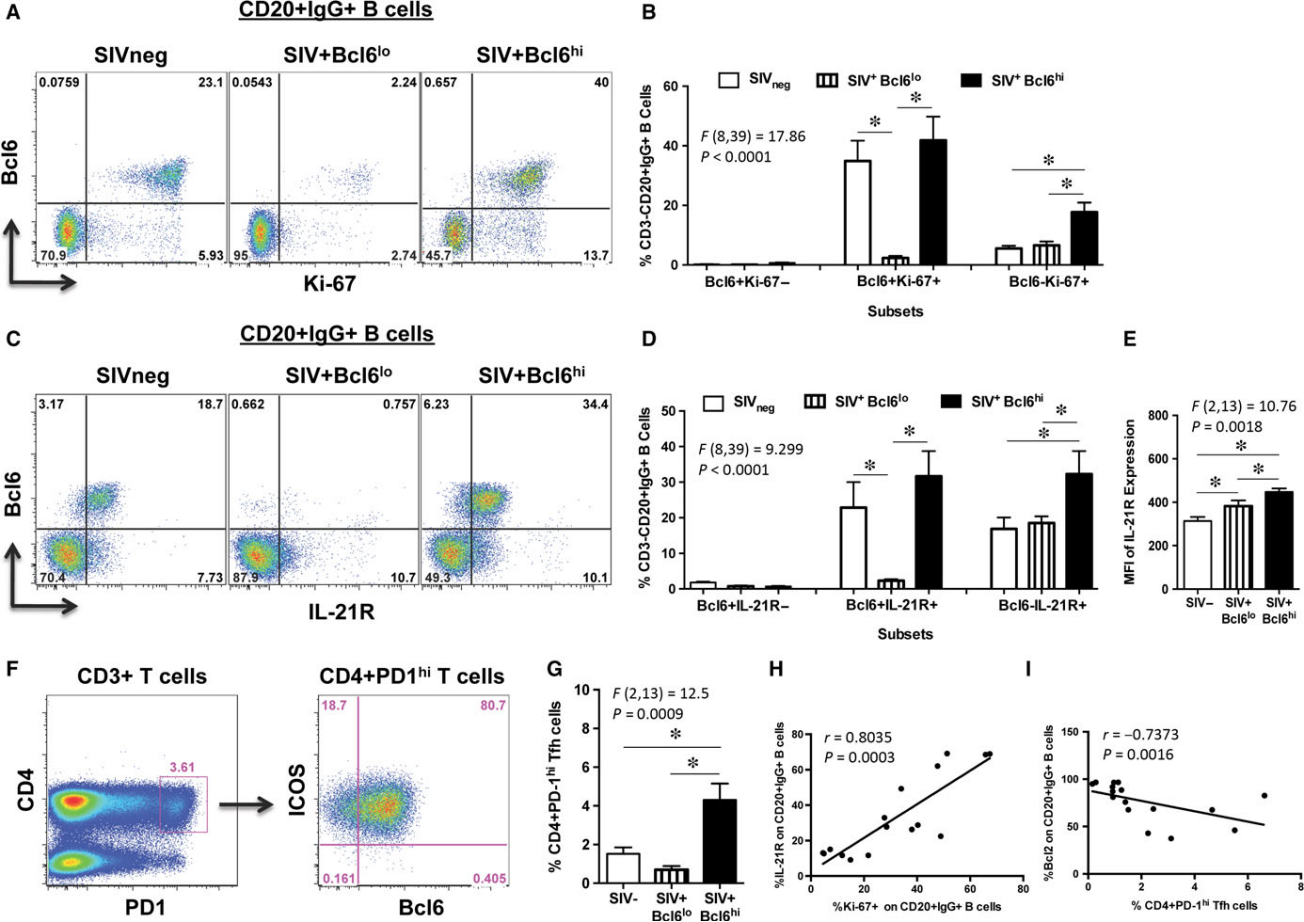
Limited expression of Ki‐67, and IL‐21R on IgG^+^ B cells in SIV^+^Bcl6^lo^ group of animals. (A) Representative dot plots showing the expression of Ki‐67 and Bcl6 on CD20^+^IgG^+^ B cells and (B) frequency of Bcl6^+^Ki‐67^‐^, Bcl6^+^Ki‐67^+^, Bcl6^‐^Ki‐67^+^ CD3^‐^CD20^+^IgG^+^ B cells in lymph nodes from SIV_neg_ (n = 6), SIV^+^Bcl6^lo^ (n = 5) and SIV^+^Bcl6^hi^ (n = 5) groups of animals. (C) Representative dot plots showing the expression of IL‐21R and Bcl6, (D) frequency of Bcl6^+^IL‐21R^‐^, Bcl6^+^IL‐21R^+^, Bcl6^‐^IL‐21R^+^ subsets and (E) mean fluorescence intensity (MFI) of IL‐21R expression on CD20^+^IgG^+^ B cells in lymph nodes from SIV_neg_ (n = 6), SIV^+^Bcl6^lo^ (n = 5) and SIV^+^Bcl6^hi^ (n = 5) groups of animals. (F) Representative dot plots showing the expression PD‐1 on CD3^+^CD4^+^ T cells and ICOS and Bcl6 expression on CD3^+^CD4^+^PD‐1^hi^ T cells to delineate Tfh cells and (G) frequency of CD3^+^CD4^+^PD‐1^hi^ Tfh cells in lymph nodes from SIV_neg_ (n = 6), SIV^+^Bcl6^lo^ (n = 5) and SIV^+^Bcl6^hi^ (n = 5) groups of animals. (H) Correlation between %IL‐21R and Ki‐67 expression on CD20^+^IgG^+^ B cells and (I) %Bcl2 on CD20^+^IgG^+^ B cells and CD4^+^PD‐1^hi^ Tfh cells from SIV_neg_ (n = 6), SIV^+^Bcl6^lo^ (n = 5) and SIV^+^Bcl6^hi^ (n = 5) groups of animals

Studies have shown that Bcl6 is critical for the differentiation and proliferation of GC B cells.^6^ To determine if the proliferative capacity of B cells differed between the groups, we examined the expression of Ki‐67 on CD20^+^IgG^+^ LN B cells from SIV^+^Bcl6^hi^ and SIV^+^Bcl6^lo^ group of animals and compared them to uninfected animals (Figure 2A). Higher levels of Bcl6 expression were associated with significantly higher frequencies of Ki‐67^+^ LN B cells in SIV^+^Bcl6^hi^ group of animals (Figure 2B) as compared to the SIV^+^Bcl6^lo^ group of animals.

Previous studies have shown that IL‐21 is a critical effector cytokine that is primarily produced by Tfh cells within the GC microenvironment that binds to IL‐21R expressed on B cells and plays a major role in driving the proliferation, differentiation and class switching of GC B cells.^12^, ^13^ To determine if the higher proliferative capacity of B cells in SIV^+^Bcl6^hi^ animals were associated with higher levels of IL‐21R expression, we examined the frequency and density of IL‐21R expression on CD20^+^IgG^+^ LN B cells. Our results showed that IL‐21R was predominantly co‐expressed with Bcl6 on B cells (Figure 2C), and the frequency and density of IL‐21R expression on B cells were significantly higher in SIV^+^Bcl6^hi^ group of animals as compared to the SIV^+^Bcl6^lo^ animals (Figure 2 D and E).

Both chronic HIV and SIV infections have been reported to exhibit an increase in Tfh cells that was found to be associated with hyperactive GC B cells and hypergammaglobulinemia.^1^, ^3^, ^4^, ^14^, ^15^, ^16^ To assess if either the increased expression or lack of Bcl6 was accompanied by changes in the frequency of Tfh, we examined the frequency of Tfh in the LN from both SIV^+^Bcl6^hi^ and SIV^+^Bcl6^lo^ group of animals and compared them to uninfected animals. Tfh cells were discriminated based on the expression of PD‐1, Bcl6 and ICOS (Figure 2F) as reported previously.^12^, ^17^, ^18^, ^19^, ^20^ Our results showed that SIV^+^Bcl6^hi^ group of animals had significantly higher frequencies of Tfh cells as compared to SIV^+^Bcl6^lo^ and SIV‐uninfected animals (Figure 2G). There was a significant positive correlation between Tfh cells and IL‐21R expression on IgG^+^ LN B cells (*r* = 0.6147, *P* = 0.0130; Figure  S1A), and between IL‐21R expression and Ki‐67^+^IgG^+^ LN B cells (*r* = 0.8035, *P* = 0.0003; Figure  2H) suggesting that IL‐21 produced by Tfh cells may have contributed to the expansion of GC B cells as earlier studies have reported.^2^, ^4^ In contrast, Tfh cells (*r* = 0.7373, *P* = 0.0016; Figure 2I) and IL‐21R expression (*r* = 0.6147, *P* = 0.0130; Figure  S1B) in SIV^+^Bcl6^lo^ group of animals negatively correlated with Bcl2 expression on IgG^+^ B cells suggesting that low levels of Tfh likely contributed to the lack of Bcl6 expression in these B cells and consequently to a higher expression of Bcl2. In line with this argument, we observed a significant positive correlation between Tfh cells and Bcl6 expression on IgG^+^ B cells (*r* = 0.6676, *P* = 0.0029; Figure  S1C). Previous studies have shown that mice lacking BCL6 fail to form GC reactions.^10^, ^21^ There was no correlation between plasma viral loads and either Tfh cells or Bcl6 expression on B cells (Figure  S1D and E).

## 4 DISCUSSION

B cell dysfunction is a hallmark of chronic HIV infection that has been shown to persist even during suppressive highly active anti‐retroviral therapy. Dysfunctional responses is thought to be driven by an expansion of Tfh cells that likely contribute to hyper‐reactive GC reactions characterized by an expansion of GC B cells that express Bcl6 and hypergammaglobulinemia.^1^, ^3^, ^4^, ^14^, ^15^, ^16^ In contrast to hyper‐reactive B cell responses, B cell exhaustion associated with chronic immune activation has been reported during chronic HIV infection.^22^ We observed similar contrasting phenotypes in chronically infected rhesus macaques with significantly higher frequencies of Bcl6^+^IgG^+^ GC B cells in SIV^+^Bcl6^hi^ animals that positively correlated with Tfh cells, whereas the SIV^+^Bcl6^lo^ group of animals had little or no Bcl6 expression but had significantly higher levels of Bcl2 expression. Interestingly, SIV^+^Bcl6^lo^ group of animals had significantly lower frequencies of Tfh cells suggesting that the lack of Tfh cells likely contributed to the lack of Bcl6 + GC B cells in these animals. Previous studies have shown that Tfh cells within the GC microenvironment drive the expression of Bcl6 in B cells.^23^ As such, we observed a significant negative correlation between Tfh cells and Bcl2 expression on LN B cells.

Higher levels of Bcl2 expression were accompanied by the lack of Ki‐67 and IL‐21R expression on B cells in the SIV^+^Bcl6^lo^ group of animals suggesting that B cell dysfunction was associated with hypo‐proliferative B cell responses. Moir et al^24^ showed that B cells from HIV‐infected patients with high plasma viremia displayed defective proliferative responses to stimuli. On the other hand, Pallikuth et al^25^ showed that HIV‐infected subjects who responded to H1N1 vaccine had higher levels of IL‐21R expressing B cells as compared to non‐responders. In fact, Ki‐67 expression on GC B cells positively correlated with IL‐21R expression suggesting that the lack of IL‐21 associated with low frequencies of Tfh cells in SIV^+^Bcl6^lo^ group of animals likely contributed to the hypo‐proliferative nature of B cells. Numerous studies have reported that Bcl6 is topographically restricted to B cells in GC in human lymphoid tissues, and inter‐ and intrafollicular CD4 T cells but not in other cells such as the mantle‐zone B cells, plasma cells, dendritic cells and macrophages^6^, ^10^ suggesting that B cell dysfunction in SIV^+^Bcl6^lo^ group of animals is likely characterized by the expansion of B cells that are outside the GC. It is unclear why some animals had an expanded Tfh phenotype while others have a low Tfh phenotype. There were no correlation between plasma viral loads and either Tfh cells or Bcl6 expression on B cells suggesting that the changes we observed may be likely related to the local LN microenvironment. It is possible that Type I IFN may play a role as some studies have suggested^26^, ^27^ and elevated levels of IFNα have been reported in the LN during SIV and HIV infections.^28^, ^29^ Additional studies are needed to delineate these changes in detail and to determine the consequences they have for immune responses.

Taken together, our results provide additional insights into the nature of B cell dysfunction during chronic HIV infection that has implications for the development of B cell immune responses against various pathogens.

## CONFLICTS OF INTEREST

The authors confirm that there are no conflicts of interest.
