# Supplementary figures and images for: Chronic simian immunodeficiency virus infection is associated with contrasting phenotypes of dysfunctional Bcl6+ germinal center B cells or Bcl6−Bcl2+ non‐germinal center B cells
Source: J Cell Mol Med. 2018 Sep 6;22(11):5682–7. doi: 10.1111/jcmm.13844 (PMC6201227; doi:10.1111/jcmm.13844)

a)

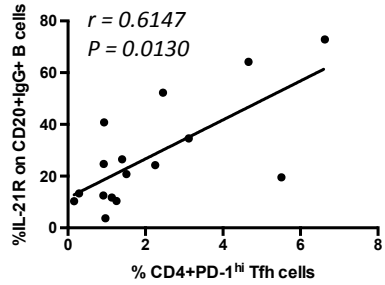

b)

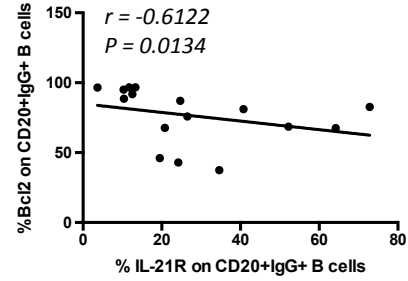

c)

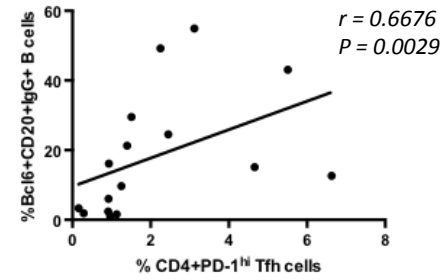

d)

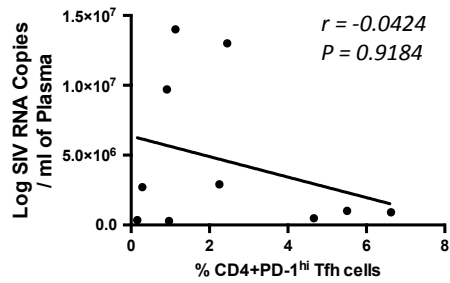

e)

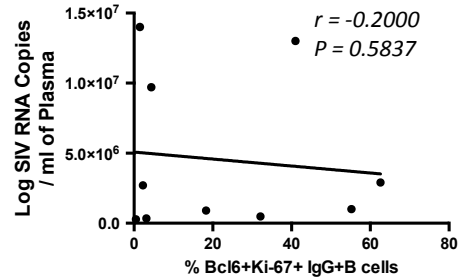

Supplement: Supplementary file 1 [file JCMM-22-5682-s001.pdf]
